# Pulsed Radiofrequency Treatment for Trigeminal Neuralgia

**DOI:** 10.5812/aapm.3493

**Published:** 2012-04-01

**Authors:** Nicholas Hai Liang Chua, Willy Halim, Tjemme Beems, Kris CP Vissers

**Affiliations:** 1 Department of Anesthesiology, Intensive Care and Pain Medicine, Tan Tock Seng Hospital, Singapore; 2 Department of Anesthesiology and Pain Management, St Anna Hospital, Geldrop, The Netherlands; 3 Department of Anesthesiology, Pain and Palliative Medicine, Radboud University, Nijmegen Medical Center, Nijmegen, The Netherlands

**Keywords:** Trigeminal Neuralgia, Trigeminal Ganglion, Trigeminal Nerve, Pulsed Radiofrequency Treatment, Minimally Invasive, Pain

## Abstract

**Background:**

Pulsed radiofrequency (PRF) treatment is defined as the delivery of short pulses of radiofrequency via a needle tip, which does not result in an actual thermal lesions. There are mixed views regarding the use of PRF for trigeminal neuralgia (TN). In our opinion, one of the main reasons for the contrasting views is the insufficient PRF dose employed in previous studies. In a recent study on the effects of PRF on resiniferatoxin-induced neuropathic pain in an animal model, the anti-allodynic effects of PRF were significantly greater when the PRF exposure duration was increased from 2 to 6 minutes.

**Objectives:**

The primary objective of this retrospective study is to report the results for 36 consecutive patients who underwent PRF treatment for TN, for 6 minutes at 45 V at a pulsed frequency of 4 Hz and a pulse width of 10 ms.

**Patients and Methods:**

For the study, we obtained procedural records of 36 consecutive patients. Their current state of pain was evaluated over a telephonic survey and the post-procedural data at 2, 6, and 12 months were retrieved thereafter from the patient records. The main outcome measure was excellent pain relief (more than 80%), which was assessed at 2, 6, and 12 months.

**Results:**

The percentages of patients who showed excellent pain relief (> 80% pain relief) at 2, 6, and 12 months were 73.5% (25/34), 61.8% (21/34), and 55.9% (19/34), respectively. The percentages of patients showing satisfactory pain relief (50–80% pain relief) at 2, 6, and 12 months were 14.7% (5/34), 17.6% (6/34), and 17.6% (6/34), respectively, and those of patients showing less than satisfactory pain relief (< 50% pain relief) at 2, 6, and 12 months were 11.8% (4/34), 20.6% (7/34), and 23.5% (8/34), respectively. No complications were reported, and none of the patients required hospitalization.

**Conclusions:**

PRF of the trigeminal ganglion should be further evaluated as an alternative treatment method for TN.

## 1. Background

The International Headache Society classifies trigeminal neuralgia (TN) into classical and symptomatic TN, with the latter being clinically indistinguishable from the former. The only identifiable difference between the 2 conditions is that in symptomatic TN, a causative lesion (other than vascular compression) can be detected, and has been demonstrated in imaging or posterior fossa exploration (International Classification of Headache Disorders-II) (1). In clinical practice, 2 phenotypic forms of TN are usually recognized, typical and atypical TN (2–4). The hallmark of typical TN is paroxysmal pain, which is lancinating in nature and occurs unilaterally in a trigeminal distribution (5). Paroxysmal pain is present in atypical TN as well, but patients often report it along with diffuse and chronic pain, which persist beyond the duration of a typical paroxysm, in the same trigeminal distribution areas. The paroxysmal pain distinguishes atypical TN from persistent idiopathic facial pain, which was previously known as atypical facial pain (1).

Carbamazepine is the drug of choice in the initial treatment of idiopathic TN. However, some patients develop adverse effects while some others do not show sustained pain relief (5). For cases in which conservative treatment is not successful, invasive treatment can be considered. The available options include surgical microvascular decompression (MVD) (6, 7), surgical sectioning of a portion of the sensory component of the trigeminal nerve, stereotactic radiation therapy or gamma knife treatment (8), percutaneous balloon microcompression (9), percutaneous glycerol rhizolysis (10), and percutaneous radiofrequency (RF) thermocoagulation of the Gasserian ganglion (11). In addition to the operative risks inherent in surgical techniques, all neurodestructive methods present risks of sensory loss, dysesthesia, anesthesia dolorosa, corneal anesthesia, and facial muscle weakness (12, 13).

Pulsed radiofrequency (PRF) treatment is defined as the delivery of short pulses of RF via a needle tip, thereby avoiding thermal lesions. This technique had been performed for various other conditions and has been shown to be effective and safe. There are contrasting opinions regarding the use of PRF treatment for TN, (14, 15) but in our opinion, one of the main reasons for this discrepancy is the insufficient PRF dose used in most studies.

## 2. Objectives

In a recent study on the effects of PRF on resiniferatoxin-induced neuropathic pain in an animal model, the anti-allodynic effects of PRF were significantly greater when the PRF exposure duration was increased from 2 to 6 minutes (16). We present a retrospective study of 36 patients with TN who underwent PRF treatment of the trigeminal ganglion for 6 minutes at 45 V, pulse frequency of 4 Hz, and pulse width of 10 ms.

## 3. Patients and Methods

### 3.1. Subjects

Institutional research review board approval was obtained prior to the retrospective collection of patient data. All patients presenting at our hospital with refractory facial neuralgia undergo a multidisciplinary assessment, including complete neurological evaluation and magnetic resonance imaging (MRI). This retrospective study included all 36 patients who underwent lateral trigeminal ganglion PRF treatment for typical and atypical TN at a single pain centre from January 2007 to April 2009. All the therapeutic procedures were performed by 2 pain physicians at our pain centre. A referring neurologist excluded secondary causes of the pain after studying MRI reports. All 36 patients presented with lancinating, burning, or aching unilateral severe facial pain, in one or more of the trigeminal nerve distributions; a small proportion of patients also experienced chronic background pain. Typical trigger points on the face in one or more of the trigeminal nerve distributions were observed in both patients with typical and atypical TN. Distinct triggering stimuli or activities such as touch, cold wind on the face, chewing, talking, and yawning were also commonly reported. Many of the painful episodes or paroxysms lasted from minutes to hours, but the episodes rarely lasted for days. Some patients reported that initial treatment with drugs such as carbamazepine, phenytoin, or gabapentin was effective, but their pain relief was rarely sustained.

### 3.2. Procedure

**Figure 1.**
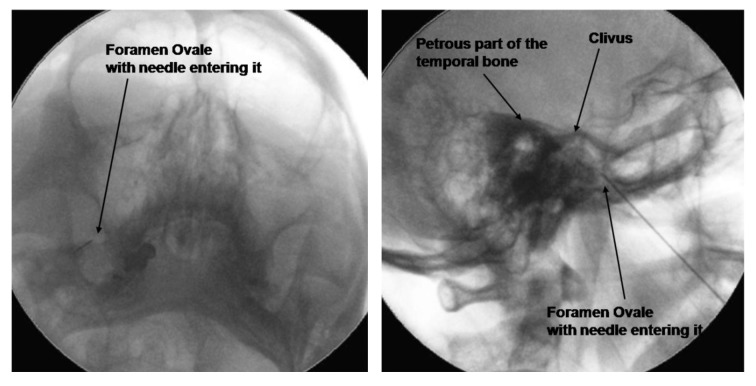
Submental View (With a 5 °Oblique Tilt) of the Foramen Ovale and Lateral View to Confirm the Depth of Needle Insertion

The percutaneous technique was performed as first described by Sweet *et al*. (11) in 1974. In this procedure, the patient lies comfortably in a supine position with the head slightly extended. Electrocardiogram and pulse oximetry and blood pressure readings are obtained for continuous hemodynamic monitoring. The C-arm is introduced in a postero-anterior fashion and rotated caudo-cranially to produce a submental view. The foramen ovale can be often already visualized with this view. A 5–10-degree tilt to the ipsilateral affected side may be required to improve visualization of the foramen ovale, as shown in *Figure 1*. The needle entry point is 2–3 cm from the corner of the mouth. An approach that worked well for us was to “bring the foramen ovale to the entry point” by manipulating the C-arm in a caudo-cranial orientation, which produced an excellent “tunnel view.”

The skin over the needle entry point is anesthetized with 1% lidocaine. Using an aseptic technique, the needle is directed towards the ipsilateral pupil. We follow the practice of keeping 1 finger in the mouth of the patient to reduce the chance of needle entry into the oral cavity. If the oral cavity is breached, the needle is replaced to reduce the rate of infectious complications. Up to 0.75 mg/kg of propofol is used to sedate the patient during the initial needle penetration into the foramen ovale. Once the needle enters the foramen ovale into Meckel’s cavity, the C-arm is then rotated laterally to ascertain the depth of penetration. The final position of the needle tip is just past the angle formed by the petrosal ridge of the temporal bone and the clivus. The propofol sedation is discontinued, the patient is allowed to awaken, and sensory stimulation is carried out at 50 Hz. The definitive position of the electrode was verified by inducing paresthesia with sensory stimulation between 0.1–0.3 V in the affected painful area. PRF is then applied for 6 minutes at 45 V, with a pulse width of 10 ms and a pulse frequency of 4 Hz. The cut-off needle tip temperature was set at 42 °C.

### 3.3. Patient Data Collection

In March 2010, an assistant attempted to contact all the 36 patients who had undergone lateral trigeminal ganglion PRF application for typical and atypical TN, in a single pain centre from January 2007 to April 2009, to enquire about their current status. After the telecommunication process was completed, retrospective patient data were retrieved from individual patient records. The perceived effect for each patient was recorded in the form of a Likert scale as a part of our routine clinic follow-up intervals at 2 months, 6 months, and 12 months. Perceived effect was recorded as a) less than 50% relief; b) 50–80% pain relief; c) more than or equal to 80% pain relief. The data entry was performed by another assistant who was not involved in the design of the study or in the analysis of the data. Descriptive statistics were generally reported as mean ± SD. Frequency counts were used to summarize categorical data. All statistical analyses were performed using the SPSS software package for Windows (version 16.0).

## 4. Results

**Figure 2.**
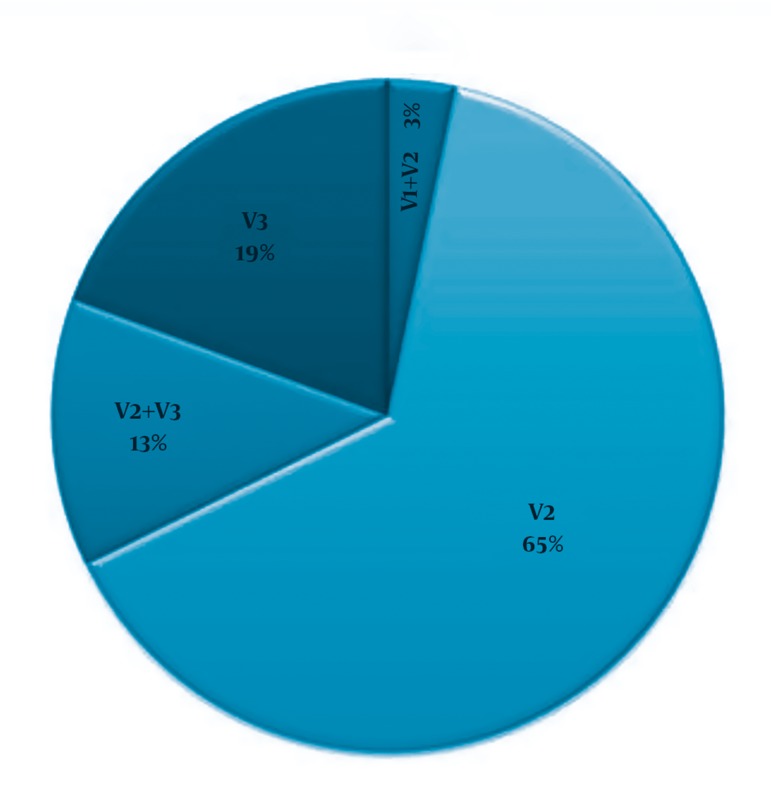
Distribution of Affected Trigeminal Branches

The pain centre procedural records showed that 36 patients had undergone PRF treatment on the trigeminal ganglion from January 2007 to January 2009. A mean duration of 2.3 ± 0.8 years have elapsed since the last PRF procedure in this group of patients, of which 67.6% still reported satisfactory pain relief. Of these 36 patients, 1 died and 1 underwent a neurosurgical procedure soon after the PRF and was unwilling to participate in the evaluation. The remaining 34 patients consented to the use of their retrospective data for analysis. The baseline characteristics of the 34 patients are shown in *Table 1*. The distribution of the affected trigeminal branches is shown in *Figure 2*.

From the retrospective review of the documented clinical results of all 34 patients, the percentages of patients who showed excellent pain relief (≥ 80% pain relief) at 2, 6, and 12 months were 73.5% (25/34), 61.8% (21/34), and 55.9% (19/34), respectively. The percentages of patients with satisfactory pain relief (50–80% pain relief) at 2, 6, and 12 months were 14.7% (5/34), 17.6% (6/34), and 17.6% (6/34), respectively, and those of patients showing less than satisfactory pain relief (< 50% pain relief) at 2, 6, and 12 months were 11.8% (4/34), 20.6% (7/34), and 23.5% (8/34), respectively. No complications were reported, and none of the patients required hospitalization.

**Table 1.** Baseline Characteristics of Patients Who Underwent Trigeminal Ganglion PRF a

Baseline Characteristics	Patients, (n = 34)
Gender, No. (%)	
Males	11 (32.4)
Females	23 (67.6)
Age, y, Mean ± SD	73 ± 14
Duration of pain, y, Mean ± SD	7.2 ± 6.2
VAS^a^ before TG^a^ PRF^a^, Mean ± SD	8.7 ± 0.7

^a^ Abbreviations: PRF, pulsed radiofrequency; TG, trigeminal ganglion; VAS, visual analogue scale

## 5. Discussion

To our knowledge, this is the largest series of TN patients treated with PRF. Among these patients, 67.6% continued to report satisfactory pain relief after 2.3 ± 0.8 years of PRF treatment. This data correlated well with our records of excellent and satisfactory rates of pain relief at 6 and 12 months. This may mean that good pain relief at 6–12 months after trigeminal ganglion PRF treatment may predict for long-term efficacy using PRF treatment. The affected trigeminal branch distributions in all 34 patients in our series were similar to those reported in the literature (5, 17).

In a prospective case series, reported by Van Zundert *et al.* (14), 5 high-risk patients received administered PRF treatment for the trigeminal ganglion. The first 4 patients experienced excellent pain relief over an average of 17.5 months, even though 1 of them required a repeat procedure. In patient 5, despite a reduction in pain intensity and frequency, the patient received conventional RF rhizotomy of the trigeminal ganglion at another centre 5 months later, with only minimal relief. This patient was eventually referred for microvascular decompression after 26 months. Our findings for this small but well-conducted case series reinforce the potential efficacy of PRF treatment in TN.

In the largest review till date, Kanpolat *et al.* (13) reported the results for 1,600 patients who had undergone percutaneous RF trigeminal rhizotomy over a period of 25 years. The complications reported in this large study were decreased corneal reflex (5.7%), weakness and paralysis of the masseter muscle (4.1%), dysesthesia (1%), anesthesia dolorosa (0.8%), keratitis (0.6%), and temporary paralysis of the third and fourth cranial nerves (0.8%). Complications like anesthesia dolorosa, though considered rare by some, are regarded to be worse than the initial pain of TN. It was perhaps for this reason that PRF was explored as a less risky alternative. However, Erdine *et al.* (15) demonstrated in a double-blinded trial that PRF was remarkably less efficacious that conventional RF. Their results demonstrate significant pain reductions in all patients treated with conventional RF, while only 2 of the 20 patients in the PRF group experienced this level of pain relief. We wish to highlight some pertinent observations that may explain the lower efficacy in the PRF group in comparison with the efficacy in the conventional RF group in that study.

The authors in that trial used the well-accepted meticulous process of conventional RF of the trigeminal ganglion. RF thermocoagulation at 70 °C for 60 s was carried out, and the sensitivity of the affected area of the face and cornea were tested thereafter. If more than 1 branch of the TN was affected, second or more procedures were performed by repositioning the needle tip and waiting for paresthesia after each procedure. Such a meticulous process, however, was not described for PRF. It appears that they performed a PRF treatment procedure, wherein 2 bursts of 20 ms were applied for 120 s at an output of 45 V (15). Notwithstanding the different end-points of both treatments, we feel that an unfair comparison had been made with regard to 2 aspects:

1. Similar to RF, if more than 1 branch of the trigeminal nerve is affected, PRF application to other affected trigeminal distributions is equally important.
2. In our experience, PRF treatment with a pulsed width of 20 ms and frequency of 2 Hz for 2 minutes is insufficient for TN.

In the case series by Van Zundert *et al.* (14), 1 patient who required a second procedure had more than 1 trigeminal branch. Even in our retrospective study, 5 out of 34 patients (14.7%) required more than one session of PRF treatment. The reason for this could be due to the neuromodulatory mode of action of PRF, which does not produce immediate paresthesia as in RF thermocoagulation. With regards to the second point, we applied PRF at 45 V, with a pulsed width of 10 ms, and a pulsed frequency of 4 Hz for 6 minutes. This higher PRF dose has recently been validated in an animal neuropathic pain-model study whereby PRF was applied to the sciatic nerve 1 week after induced injury for 2, 4, and 6 minutes (16). The group where PRF was applied for 6 minutes showed increased *withdrawal latency-increased anti-allodynic effects*, than the groups with 2 or 4 minutes of PRF application.

A systematic review of ablative neurosurgical techniques for the treatment of TN evaluated 166 studies reporting RF thermocoagulation, glycerol rhizolysis, balloon compression of the trigeminal ganglion, and stereotactic radiosurgery and concluded that RF thermocoagulation offers the highest rates of complete pain relief (2). In our opinion, RF trigeminal rhizotomy is still an invaluable technique that has provided pain relief for many patients with TN. In our opinion, PRF needs to be performed to a similar degree to be compared in the same light. It may be prudent to even consider performing PRF prior to RF for a sole purpose of avoiding disturbing sensory paresthesia and masseter paralysis.

The limitations of this study are inherent to retrospective studies of such nature, in which data have been collected in a clinical context and cannot, for example, allow quantification of the changes in pain medications over 1 year of follow-up. PRF treatment of the trigeminal ganglion may be a possible alternative to minimally invasive treatment in the management of TN. The possibilities of reduced heat-related complications and comparable efficacies to conventional modalities need to be evaluated in greater detail in further studies.

## References

[1] (2004). The International Classification of Headache Disorders: 2nd edition.. Cephalalgia..

[2] Lopez BC, Hamlyn PJ, Zakrzewska JM (2004). Systematic review of ablative neurosurgical techniques for the treatment of trigeminal neuralgia.. Neurosurgery..

[3] van Kleef M, van Genderen WE, Narouze S, Nurmikko TJ, van Zundert J, Geurts JW (2009). 1. Trigeminal neuralgia.. Pain Pract..

[4] Burchiel KJ (2003). A new classification for facial pain.. Neurosurgery..

[5] Bennetto L, Patel NK, Fuller G (2007). Trigeminal neuralgia and its management.. BMJ..

[6] Jannetta PJ, McLaughlin MR, Casey KF (2005). Technique of microvascular decompression. Technical note.. Neurosurg Focus..

[7] Jannetta PJ, Tew JM, Jr. (1979). Treatment of trigeminal neuralgia.. Neurosurgery..

[8] Young RF, Vermulen S, Posewitz A (1998). Gamma knife radiosurgery for the treatment of trigeminal neuralgia.. Stereotact Funct Neurosurg..

[9] Mullan S, Lichtor T (1983). Percutaneous microcompression of the trigeminal ganglion for trigeminal neuralgia.. J Neurosurg..

[10] Hakanson S (1981). Trigeminal neuralgia treated by the injection of glycerol into the trigeminal cistern.. Neurosurgery..

[11] Sweet WH, Wepsic JG (1974). Controlled thermocoagulation of trigeminal ganglion and rootlets for differential destruction of pain fibers. 1. Trigeminal neuralgia.. J Neurosurg..

[12] Apfelbaum RI (1977). A comparision of percutaneous radiofrequency trigeminal neurolysis and microvascular decompression of the trigeminal nerve for the treatment of tic douloureux.. Neurosurgery..

[13] Kanpolat Y, Savas A, Bekar A, Berk C (2001). Percutaneous controlled radiofrequency trigeminal rhizotomy for the treatment of idiopathic trigeminal neuralgia: 25-year experience with 1,600 patients.. Neurosurgery..

[14] Van Zundert J, Brabant S, Van de Kelft E, Vercruyssen A, Van Buyten JP (2003). Pulsed radiofrequency treatment of the Gasserian ganglion in patients with idiopathic trigeminal neuralgia.. Pain..

[15] Erdine S, Ozyalcin NS, Cimen A, Celik M, Talu GK, Disci R (2007). Comparison of pulsed radiofrequency with conventional radiofrequency in the treatment of idiopathic trigeminal neuralgia.. Eur J Pain..

[16] Tanaka N, Yamaga M, Tateyama S, Uno T, Tsuneyoshi I, Takasaki M (2010). The effect of pulsed radiofrequency current on mechanical allodynia induced with resiniferatoxin in rats.. Anesth Analg..

[17] Siccoli MM, Bassetti CL, Sandor PS (2006). Facial pain: clinical differential diagnosis.. Lancet Neurol..

